# Supplementary material for: Identification of Signature Genes of Dilated Cardiomyopathy Using Integrated Bioinformatics Analysis
Source: Int J Mol Sci. 2023 Apr 16;24(8):7339. doi: 10.3390/ijms24087339 (PMC10139023; doi:10.3390/ijms24087339)
Supplement: Supplementary file 1 [file ijms-24-07339-s001.zip › Table S1.pdf]

Table S1. Samples information included in the datasets for this study.

GSE ID	GSM ID (Control)	GSM ID (Dilated cardiomyopathy)
GSE3585	GSM2251530<br>GSM2251531<br>GSM2251532<br>GSM2251533<br>GSM2251534	GSM2251530<br>GSM2251531<br>GSM2251532<br>GSM2251533<br>GSM2251534<br>GSM2251535<br>GSM2251536
GSE29819	GSM739016<br>GSM739018<br>GSM739020<br>GSM739022<br>GSM739024<br>GSM739026	GSM739002<br>GSM739004<br>GSM739006<br>GSM739008<br>GSM739010<br>GSM739012<br>GSM739014
GSE42955	GSM1053939<br>GSM1053940<br>GSM1053942<br>GSM1053922<br>GSM1053929	GSM1053915<br>GSM1053917<br>GSM1053918<br>GSM1053919<br>GSM1053941<br>GSM1053924<br>GSM1053925<br>GSM1053926<br>GSM1053933<br>GSM1053935<br>GSM1053937<br>GSM1053938
GSE43435	GSM1062380<br>GSM1062381<br>GSM1062382<br>GSM1062383<br>GSM1062384<br>GSM1062385<br>GSM1062386<br>GSM1062387<br>GSM1062388<br>GSM1062389	GSM1062390<br>GSM1062391<br>GSM1062392<br>GSM1062393<br>GSM1062394<br>GSM1062395<br>GSM1062396<br>GSM1062397<br>GSM1062398<br>GSM1062399
GSE79962	GSM2109150<br>GSM2109151<br>GSM2109152<br>GSM2109153<br>GSM2109154	GSM2109130<br>GSM2109131<br>GSM2109132<br>GSM2109133<br>GSM2109134

	GSM2109155<br>GSM2109156<br>GSM2109157<br>GSM2109158<br>GSM2109159<br>GSM2109160	GSM2109135<br>GSM2109136<br>GSM2109137<br>GSM2109138
GSE84796	GSM2251540<br>GSM2251541<br>GSM2251542<br>GSM2251543<br>GSM2251544<br>GSM2251545<br>GSM2251546	GSM2251530<br>GSM2251531<br>GSM2251532<br>GSM2251533<br>GSM2251534<br>GSM2251535<br>GSM2251536<br>GSM2251537<br>GSM2251538<br>GSM2251539
