# Supplementary material for: Identification of Signature Genes of Dilated Cardiomyopathy Using Integrated Bioinformatics Analysis
Source: Int J Mol Sci. 2023 Apr 16;24(8):7339. doi: 10.3390/ijms24087339 (PMC10139023; doi:10.3390/ijms24087339)
Supplement: Supplementary file 1 [file ijms-24-07339-s001.zip › Table S2.pdf]

Table S2. The 195 DEGs identified by RRA method.

88 upregulated DEGs in RRA		107 downregulated DEGs in RRA	
**Gene symbol**	**P-value**	**Gene symbol**	**P-value**
FRZB	8.53×10^{-5}	*FCN3*	4.29×10^{-6}
PHLDA1	3.32×10^{-4}	*MID1IP1*	8.19×10^{-6}
NPPA	0.001321	*CD14*	3.19×10^{-5}
NAP1L3	0.001563	*CCDC69*	3.86×10^{-5}
PLCE1	0.001628	*IMPA2*	4.84×10^{-5}
DPT	0.002045	*PTDSS1*	1.81×10^{-4}
LTBP4	0.002825	*NSG1*	1.89×10^{-4}
MFAP4	0.002886	*MYH6*	2.59×10^{-4}
USP11	0.002956	*C1orf105*	7.51×10^{-4}
HMGB2	0.003888	*PPP1R1A*	0.001422
ODC1	0.004056	*ATP2A2*	0.001547
NEB	0.004698	*OCIAD2*	0.001851
TRMT5	0.00552	*CHDH*	0.002128
IGFBP3	0.005819	*RRAD*	0.002137
SPRED2	0.006762	*ETNPPL*	0.002267
FMOD	0.006795	*S100A9*	0.002287
ZEB1	0.007092	*APOBEC2*	0.002357
CCN2	0.007455	*CCL11*	0.002452
TSC22D3	0.007685	*SERPINA3*	0.002842
SMOC2	0.007696	*STAT3*	0.002894
TNNI1	0.007696	*H1-2*	0.003053
NPPB	0.007696	*CDKN3*	0.003169
MTND1P23	0.007696	*TIMMDC1*	0.003362
IGHG3	0.007696	*CCL2*	0.003766
SOCS2	0.008487	*NAMPT*	0.003976
C10orf71	0.008522	*H2BC5*	0.004192
OMD	0.008771	*CHMP4B*	0.006522
HTRA1	0.009614	*HMOX2*	0.006522
PPDPF	0.009614	*EMD*	0.006522
PDE8B	0.009816	*TIMELESS*	0.006522
PIK3CA	0.013401	*D4S234E*	0.006522
LAMB1	0.013586	*IFRD2*	0.007133
SNCA	0.014264	*B3GALT2*	0.007133
LTBP2	0.014436	*LYVE1*	0.007503
SULF1	0.014436	*CCR1*	0.008709
BMP4	0.015385	*RETSAT*	0.010068
HMGN3	0.015385	*CCNG1*	0.01034
BEX1	0.015385	*RARRES1*	0.012046
CPED1	0.015385	*CDCP1*	0.013039
IGJ	0.015385	*IDH2*	0.013039

LRRC17	0.0155	*AVPI1*	0.013039
MYH10	0.017273	*SEMA3F*	0.013039
INPP4B	0.01779	*lincRNA:chr15:70477371-70577596_R*	0.013039
PDK4	0.01779	*GTF3A*	0.013229
SFRP4	0.019932	*MOG*	0.013229
FSTL3	0.019932	*CES2*	0.013541
PRELP	0.021615	*ASB12*	0.01521
PIK3R1	0.02219	*BCL3*	0.01521
ZFAND5	0.023065	*ST3GAL5*	0.016039
CFH	0.023065	*LPCAT3*	0.016889
DST	0.023065	*CA14*	0.017035
OGDHL	0.023065	*IFITM1*	0.018653
DYNLT1	0.023065	*FCER1G*	0.019549
IGKV3-7	0.023065	*MCM8*	0.019549
SLC16A9	0.023081	*KIAA1957*	0.019549
RGCC	0.024564	*NP450512*	0.019549
ZNF704	0.025086	*KLHDC8B*	0.019567
PCOLCE2	0.025175	*TST*	0.019567
FILIP1L	0.025175	*F13A1*	0.021456
ASPN	0.026419	*CADPS2*	0.021942
SEPTIN11	0.027886	*H1-0*	0.022928
FGF1	0.028149	*AMD1*	0.023111
ZNF189	0.028415	*RNASE2*	0.023429
LTBP1	0.029654	*STRADB*	0.023429
ZMAT1	0.030736	*CUTC*	0.023935
ABCG2	0.030736	*E2F8*	0.024963
SNORD14C	0.030736	*PDAP1*	0.026054
SLPI	0.030736	*HTATIP2*	0.026054
IGHV3-23	0.030736	*BMP4*	0.026054
RECK	0.030997	*CPAMD8*	0.026054
ECM2	0.03168	*AGXT2L1*	0.026054
KDM3A	0.032369	*SLC29A1*	0.02708
SSPN	0.032369	*ANXA3*	0.02708
POSTN	0.032876	*S100A8*	0.029278
GOLGA4-AS1	0.0384	*NKX2-5*	0.02984
MATN2	0.0384	*VSIG4*	0.030408
SNORA50A	0.0384	*SLC5A1*	0.03098
S77011	0.0384	*MFSD1*	0.03098
OGN	0.039645	*MZT2A*	0.031558
ZBTB16	0.042749	*F5*	0.031558
LTBP3	0.045149	*FAM167B*	0.032553
ROR1	0.045149	*ZNF331*	0.032553

CYP2J2	0.045962	*MTUS2*	0.032553
MTURN	0.046055	*SAMM50*	0.032553
VTRNA1-1	0.046055	*TUBA3E*	0.032553
ITLN1	0.046055	*GNMT*	0.034521
GOLIM4	0.046055	*S100A3*	0.035129
IGLC2	0.046055	*CYYR1*	0.035741
		ART3	0.036982
		KCNIP2	0.037609
		PIM3	0.037609
		GPRC5B	0.039045
		CLEC10A	0.039045
		ALOX5AP	0.039045
		RBM38	0.039045
		ADAMTS9	0.040169
		CD163	0.040169
		G0S2	0.040362
		FSD2	0.041479
		FCGBP	0.045526
		RBPMS2	0.045532
		CYB5A	0.045532
		ZNF385	0.045532
		NCKAP1L	0.046217
		DLEU1	0.047151
		PLSCR1	0.048321
		TUBA1C	0.049032
