# Supplementary material for: Identification of Signature Genes of Dilated Cardiomyopathy Using Integrated Bioinformatics Analysis
Source: Int J Mol Sci. 2023 Apr 16;24(8):7339. doi: 10.3390/ijms24087339 (PMC10139023; doi:10.3390/ijms24087339)
Supplement: Supplementary file 1 [file ijms-24-07339-s001.zip › Table S3.pdf]

Table S3. Quality control and sequencing information for samples.

Sample	Raw reads	Clean reads	Clean bases	Error(%)	Q20(%)	Q30(%)	GC(%)
C-1	56008732	56003780	8.3G	0.04	97.69	93.17	48.95
C-2	52738170	52733316	7.8G	0.04	97.59	92.89	48.88
C-3	61459346	61453442	9.09G	0.04	97.36	92.32	49.96
M-1	45194800	45190742	6.46G	0.04	97.73	93.23	54.4
M-2	50903326	50899634	7.53G	0.04	97.68	93.15	49.38
M-3	46826690	46823072	6.88G	0.04	97.76	93.34	50.71
