# Supplementary material for: Identification of Signature Genes of Dilated Cardiomyopathy Using Integrated Bioinformatics Analysis
Source: Int J Mol Sci. 2023 Apr 16;24(8):7339. doi: 10.3390/ijms24087339 (PMC10139023; doi:10.3390/ijms24087339)
Supplement: Supplementary file 1 [file ijms-24-07339-s001.zip › Table S4.pdf]

Table S4. The 362 DEGs identified by RNA-Seq.

224 upregulated DEGs in RNA-Seq		138 downregulated DEGs in RNA-Seq	
**Gene symbol**	**P-value**	**Gene symbol**	**P-value**
Scgb1a1	1.25×10^{-9}	*Lmntd1*	4.65×10^{-6}
Gnb3	7.83×10^{-7}	*Fgf12*	6.74×10^{-6}
Cnksr1	4.65×10^{-6}	*Nrep*	2.66×10^{-5}
Myh7	4.65×10^{-6}	*Gm40841*	6.00×10^{-5}
Ptprn	7.02×10^{-5}	*Lypd8l*	7.02×10^{-5}
Gnmt	8.19×10^{-5}	*Postn*	8.19×10^{-5}
Per1	0.00033	*Ccr5*	0.00033
Cyp1a1	0.000355	*Top2a*	0.000433
Gm6402	0.000521	*Cd55*	0.000504
Scx	0.000548	*Aqp4*	0.000511
Novel.2340	0.000693	*Cilp2*	0.000522
H4c8	0.001046	*Ptgfr*	0.000963
Dbp	0.001096	*Col14a1*	0.001539
Trim54	0.001236	*Ppp1r1b*	0.001573
Comt	0.001573	*Vcan*	0.001573
Dcxr	0.001687	*C7*	0.001596
Mir133a-2	0.002033	*Camk1d*	0.001687
Gm11967	0.002179	*Sytl2*	0.001687
Gm40457	0.002228	*Kcnj3*	0.002033
Myom2	0.002347	*Sphkap*	0.002078
Atcayos	0.003401	*Hcn1*	0.003204
Rasd2	0.003401	*Ccn3*	0.003401
Sftpc	0.003569	*Sbk3*	0.004189
Gm13611	0.004189	*Pi15*	0.00433
Tcap	0.00433	*Rp1*	0.005583
Sftpb	0.004783	*Lcp1*	0.005583
Epn3	0.004783	*Pla2g4a*	0.00629
Bex1	0.005258	*Itga8*	0.006553
Gm15469	0.005387	*Slc7a7*	0.006804
Mknk2	0.005583	*Adamts19*	0.006882
Psmg4	0.005583	*Ptprc*	0.006956
Rhd	0.00629	*Wif1*	0.008083
Cdhr3	0.006882	*Bmp3*	0.008415
Novel.24909	0.007495	*E030013I19Rik*	0.0088
Pnpla2	0.007872	*Cd209f*	0.0088
Novel.16295	0.007888	*Fstl1*	0.008959
Scgb1c1	0.0088	*Itih5*	0.009025
Ndufa2	0.0088	*Tspan6*	0.009025
Prxl2b	0.009067	*Tmem163*	0.009053
Klk1b26	0.009714	*Gm32849*	0.009714

Etv5	0.010165	*Tspan2*	0.010278
Plin3	0.010165	*Tbc1d1*	0.010278
Rps15-ps3	0.010165	*Novel.11634*	0.010278
Polr2e	0.0109	*Sertad4*	0.0109
Cirbp	0.01132	*Aspn*	0.0109
Bola1	0.011512	*Scd1*	0.0109
Gm19439	0.011512	*Cd34*	0.011292
Cdkn1a	0.012447	*Aldh3b3*	0.01132
Rpl36-ps12	0.013204	*Sema3c*	0.011564
Mgst3	0.01399	*Mki67*	0.01165
Psmd4	0.014091	*Sbk2*	0.012076
Rpl36	0.014091	*Pitx2*	0.012236
Cst6	0.014091	*Aldh1l2*	0.012336
Rpl36-ps2	0.014532	*Gm20559*	0.012397
Eif1-ps1	0.01548	*Rgs7*	0.014091
Smim4	0.015852	*Iqgap3*	0.014091
Lrg1	0.015852	*Gm33543*	0.014091
Novel.1566	0.016354	*Gm48898*	0.015035
Lcn2	0.017163	*Gask1b*	0.015852
Gm11808	0.017163	*Cacna2d2*	0.015852
Gm10073	0.017455	*Pck1*	0.017163
Gm11730	0.017455	*Ccna2*	0.017455
Novel.11952	0.017455	*Lum*	0.017573
Rplp1	0.017573	*Psd3*	0.017696
Eif4ebp1	0.017696	*Abi3bp*	0.017702
Romo1	0.017872	*Dkk3*	0.019223
1500002F19Rik	0.017872	*Tmem45b*	0.019503
Slc26a10	0.017872	*Timd4*	0.01964
Novel.21540	0.017872	*Dpt*	0.021583
Gm49123	0.017881	*Cdcp3*	0.021583
Novel.18074	0.018102	*Angpt1*	0.021583
Bola2	0.01815	*Myof*	0.022229
Novel.24937	0.018647	*Fras1*	0.022278
Tcf23	0.018817	*Gm28979*	0.023722
Rps11-ps1	0.019048	*Mrc1*	0.023963
Gm15427	0.01941	*Alox8*	0.024954
Yjefn3	0.01941	*Dzip3*	0.026172
BB123696	0.019692	*Tfrc*	0.026172
Srarp	0.021583	*Gja5*	0.026223
Sema3b	0.02321	*Mybphl*	0.02632
C1qtnf4	0.023603	*Itgb8*	0.02632
Lrrc10	0.023603	*Gm12522*	0.026497
Gm5436	0.02369	*Olfm1*	0.027076

Timp4	0.023722	*Plekha7*	0.027076
Mif	0.023722	*Sort1*	0.027112
Gm6581	0.023722	*Igfbpl1*	0.027524
Tnnc1	0.023805	*Fam111a*	0.028173
Gm11942	0.023938	*Tpm4*	0.028411
Proser2	0.024954	*Ecm2*	0.030369
Rplp2	0.026172	*Mfap4*	0.032024
Car4	0.026172	*Cdk1*	0.032029
Plin4	0.026172	*Sln*	0.032073
Pla2g12a	0.026709	*Smc2*	0.033612
Gck	0.026709	*Car3*	0.034317
Pex16	0.027076	*Meox1*	0.035157
A530013C23Rik	0.027076	*Gm13054*	0.035866
Gm7331	0.027076	*Cxcl13*	0.035866
Depp1	0.027076	*Vat1l*	0.035866
Use1	0.027076	*Lcp2*	0.035866
Novel.7907	0.027076	*Map1b*	0.035866
Novel.23522	0.027076	*Cpne5*	0.035866
Mybpc3	0.027242	*Scd2*	0.035866
Gm14303	0.027242	*Mme*	0.036466
Rps13	0.027242	*Dab2*	0.036466
Atg101	0.027315	*Myl1*	0.036508
Gm5781	0.027524	*Rian*	0.036859
Tuba1c	0.028173	*Sfrp5*	0.037148
H2aj	0.028585	*A2m*	0.037758
Mt1	0.028585	*Mgl2*	0.037758
Rpsa-ps2	0.029443	*Alox15*	0.037758
Hrct1	0.030044	*Ccr2*	0.03796
Novel.16760	0.030044	*Lingo1*	0.037967
Des	0.030583	*Slc5a3*	0.03965
Novel.3073	0.030584	*Armcx1*	0.03991
Cebpb	0.031778	*Arl5a*	0.040531
Ndufb7	0.032024	*Efhd1*	0.041003
Gm15417	0.032073	*Rxfp1*	0.041003
Gm5805	0.032073	*Slc26a3*	0.041056
Gm2810	0.03256	*Adipoq*	0.041056
Rpl18a	0.032813	*Tmem35a*	0.041457
Adprhl1	0.033347	*Elovl6*	0.041457
Hspb7	0.03336	*Rps6ka5*	0.041457
Ndufs7	0.03337	*Ddr2*	0.041716
Per2	0.033612	*Alas2*	0.042455
Rpsa-ps9	0.033612	*Opcml*	0.042491
Gm13226	0.033612	*Mpp7*	0.043716

Gm5617	0.033612	*Acvr1c*	0.044028
Tmem205	0.033612	*Vsig4*	0.044486
Rps12-ps24	0.03457	*Snca*	0.0457
Gm10138	0.035866	*Slc17a7*	0.045847
Paxx	0.035866	*Plagl1*	0.045847
Lsm10	0.035866	*Kcnj15*	0.046527
Tbx3os1	0.035866	*Tlcd4*	0.048016
Rps4l	0.035866	*Gprin3*	0.048016
Gm44020	0.035866	*Lgals12*	0.048564
Tmem160	0.035866	*Ccn4*	0.048576
Lgals4	0.035866	*Col15a1*	0.048932
Rabac1	0.035866	*Sh3pxd2b*	0.049788
Ccdc124	0.035866		
Gm30556	0.035866		
Anapc13	0.035866		
Fstl4	0.035866		
Sgca	0.035866		
Dennd6b	0.035866		
Prodh	0.035866		
Slc25a28	0.035866		
Novel.5898	0.035866		
Novel.20876	0.035866		
Novel.20655	0.035866		
Sult1a1	0.036277		
Upp1	0.036466		
Novel.17404	0.036466		
Atp6ap1l	0.037549		
Gm6155	0.037758		
5430431A17Rik	0.037758		
Prmt1	0.037758		
Gm17823	0.037758		
Rgcc	0.037758		
Ndufb9	0.037758		
Gm9843	0.037758		
Uqcc3	0.037758		
Novel.13562	0.037758		
Snta1	0.037831		
Rps15	0.037831		
Gdf15	0.03796		
Lsm4	0.03796		
Myl3	0.03796		
Rps10-ps1	0.038196		
Clec18a	0.038196		

ENSMUSG0000207555 1	0.038196		
Novel.19232	0.038853		
Snhg8	0.038958		
Pgls	0.038958		
Cdh4	0.039044		
Atp5e	0.040307		
Rpsa-ps10	0.040307		
Gpx4	0.040307		
Sema6b	0.040307		
Ager	0.040307		
Novel.9012	0.040307		
Rpl37rt	0.040531		
Novel.13148	0.040531		
Zfp775	0.041457		
Gm19810	0.041457		
Misp3	0.041457		
Snai3	0.041457		
Gm4708	0.041457		
Atp5k-ps2	0.042455		
Tspan4	0.042455		
Emc9	0.042455		
Ptp4a3	0.042524		
Adgrg1	0.043311		
Uqcrq	0.043569		
Gm33424	0.043569		
Rps28	0.043618		
Fau	0.043749		
Rpl37	0.043814		
Sod1	0.043814		
Polr2l	0.043992		
Novel.22195	0.043992		
Clpp	0.044486		
1810044D09Rik	0.044516		
Gpx4-ps2	0.044641		
Nqo1	0.044775		
Rps14	0.045349		
Tbcb	0.0457		
Catsper4	0.045831		
Gm9294	0.046776		
Dohh	0.046844		
Gm8566	0.047891		
Gm4950	0.047916		

Novel.9845	0.048016		
Gm15501	0.048393		
Ccs	0.048393		
Hint2	0.048425		
Nat9	0.048425		
Rpl38	0.048564		
Neurl2	0.048576		
Sympk	0.048576		
Gm6576	0.048741		
Kcnc1	0.04907		
Cd63	0.04907		
Shld1	0.049446		
Fbxo31	0.049788		
