# Supplementary material for: Identification of Signature Genes of Dilated Cardiomyopathy Using Integrated Bioinformatics Analysis
Source: Int J Mol Sci. 2023 Apr 16;24(8):7339. doi: 10.3390/ijms24087339 (PMC10139023; doi:10.3390/ijms24087339)
Supplement: Supplementary file 1 [file ijms-24-07339-s001.zip › Table S5.pdf]

Table S5. Enrichment analysis results of RNA-Seq differential genes.

GO analysis of DEGs associated with DCM in the RNA-Seq analysis				KEGG analysis of DEGs associated with DCM in the RNA-Seq analysis			
**ID**	**Description**	**Gene count**	**P-value**	**ID**	**Description**	**Gene count**	**P-value**
GO:0005198	structural molecule activity	29	1.90E-09	mmu05410	Hypertrophic cardiomyopathy	10	1.81E-06
GO:0007275	multicellular organism development	109	1.73E-08	mmu05414	Dilated cardiomyopathy	10	2.45E-06
GO:0032502	developmental process	123	1.85E-08	mmu03010	Ribosome	13	3.70E-06
GO:0048856	anatomical structure development	117	1.52E-08	mmu05171	Coronavirus disease - COVID-19	14	3.66E-05
GO:0051239	regulation of multicellular organismal process	78	7.29E-09	mmu04260	Cardiac muscle contraction	6	0.002725
GO:0022626	cytosolic ribosome	12	1.52E-08	mmu05016	Huntington disease	12	0.003337
GO:0009888	tissue development	53	4.53E-08	mmu05208	Chemical carcinogenesis - reactive oxygen species	10	0.002977
GO:0048731	system development	99	6.46E-08	mmu04152	AMPK signaling pathway	7	0.004169
GO:0070887	cellular response to chemical stimulus	69	2.54E-07	mmu05412	Arrhythmogenic right ventricular cardiomyopathy	5	0.007897
GO:0006636	unsaturated fatty acid biosynthetic process	8	4.92E-07	mmu04723	Retrograde endocannabinoid signaling	7	0.009523
GO:0050727	regulation of inflammatory response	19	6.34E-07	mmu05012	Parkinson disease	10	0.009919
GO:0070848	response to growth factor	26	6.73E-07	mmu05020	Prion disease	10	0.010956
GO:0030198	extracellular matrix organization	16	7.59E-07	mmu00590	Arachidonic acid metabolism	5	0.012414
GO:0043062	extracellular structure organization	17	1.23E-06	mmu03320	PPAR signaling pathway	5	0.014242
GO:0051240	positive regulation of multicellular organismal process	49	1.62E-06	mmu01040	Biosynthesis of unsaturated fatty acids	3	0.017013
GO:0003735	structural constituent of ribosome	12	1.69E-06	mmu00190	Oxidative phosphorylation	6	0.021021
GO:0042592	homeostatic process	48	1.91E-06	mmu04713	Circadian entrainment	5	0.020793
GO:0048513	animal organ development	75	2.59E-06	mmu05010	Alzheimer disease	12	0.019839
GO:0031012	extracellular matrix	20	3.46E-06	mmu00350	Tyrosine metabolism	3	0.024518
GO:0008015	blood circulation	20	4.03E-06	mmu04216	Ferroptosis	3	0.026201
GO:0065008	regulation of biological quality	80	4.25E-06	mmu00910	Nitrogen metabolism	2	0.029923
GO:1901681	sulfur compound	14	4.17E-06	mmu04066	HIF-1 signaling pathway	5	0.035576

	binding						
GO:0003013	circulatory system process	20	5.76E-06	mmu04261	Adrenergic signaling in cardiomyocytes	6	0.036002
GO:0016043	cellular component organization	111	5.92E-06	mmu05014	Amyotrophic lateral sclerosis	11	0.034556
GO:0005737	cytoplasm	174	5.92E-06	mmu04932	Non-alcoholic fatty liver disease	6	0.039029
GO:0005201	extracellular matrix structural constituent	10	6.43E-06	mmu05022	Pathways of neurodegeneration - multiple diseases	13	0.039507
GO:0031099	regeneration	13	7.09E-06	mmu00360	Phenylalanine metabolism	2	0.048246
GO:0044391	ribosomal subunit	12	8.03E-06	mmu00591	Linoleic acid metabolism	3	0.046319
GO:0044444	cytoplasmic part	145	8.00E-06	mmu04514	Cell adhesion molecules	6	0.050269
GO:0005840	ribosome	13	8.52E-06	mmu04964	Proximal tubule bicarbonate reclamation	2	0.048246
